# Pulsatile Culprits: Two Cases of Vertebral Artery Medullary Compression Syndrome

**DOI:** 10.7759/cureus.110157

**Published:** 2026-06-03

**Authors:** Nikhil Arora, Pooja Sethi, Rose Mary Jose

**Affiliations:** 1 Radiodiagnosis, Government Medical College and Hospital, Aurangabad, Aurangabad, IND; 2 Radiology, Manipal Hospitals, Bengaluru, IND; 3 Radiology, Government Medical College and Hospital, Aurangabad, Aurangabad, IND

**Keywords:** medulla compression, neurovascular compression syndrome, vertebral artery medullary compression syndrome, vertebral artery variant, vertebrobasilar dolichoectasia

## Abstract

Vertebral artery medullary compression syndrome (VAMCS) is an uncommon neurovascular condition caused by tortuous or ectatic vertebral arteries producing focal compression of the medulla oblongata or cervicomedullary junction. Clinical manifestations are heterogeneous and may include vertigo, imbalance, ataxia, dysphagia, dysarthria, pyramidal tract signs, sensory disturbances, and limb weakness, depending on the site and severity of compression. Because vertebral artery indentation of the medulla may also occur incidentally in asymptomatic individuals, diagnosis requires clinical correlation and exclusion of alternative pathologies.

We present two cases demonstrating different scenarios of VAMCS. The first case involved a 77-year-old woman presenting with acute left-sided hemiparesis. MRI demonstrated acute infarcts involving the right insular and occipital cortex and right corona radiata, along with chronic ischemic changes. Additional imaging revealed a tortuous and mildly ectatic dominant left vertebral artery, causing focal compression of the left anterolateral medulla. The coexistence of ischemic infarcts represented a confounding factor in differentiating symptomatic neurovascular compression from concurrent cerebrovascular disease.

The second case involved a 46-year-old man presenting with chronic right-sided limb weakness, imbalance, and ataxia. MRI demonstrated focal compression of the medulla by a prominent left vertebral artery and posterior inferior cerebellar artery branch without associated intrinsic medullary signal abnormality. No acute infarct or alternative posterior fossa pathology was identified. In the absence of competing structural lesions, the findings were considered consistent with isolated symptomatic VAMCS.

These cases emphasize the importance of evaluation of the cervicomedullary junction on MRI, especially in patients with unexplained posterior fossa or pyramidal tract symptoms. High-resolution MRI and MR angiography play a critical role in demonstrating neurovascular compression and excluding alternative diagnoses. Greater awareness of this uncommon entity may help improve recognition and avoid underdiagnosis.

## Introduction

Vertebral artery medullary compression syndrome (VAMCS) is an uncommon neurovascular condition caused by tortuous or ectatic vertebral arteries producing focal compression of the medulla or cervicomedullary junction [1,2]. It is considered part of the broader spectrum of neurovascular compression syndromes and is most commonly related to vertebrobasilar dolichoectasia, although symptomatic medullary compression remains underrecognized due to its heterogeneous and nonspecific clinical presentation [1-3]. Reported manifestations include vertigo, imbalance, ataxia, dysphagia, dysarthria, limb weakness, sensory disturbances, and pyramidal tract signs depending on the site and severity of compression [2-5].

Magnetic resonance imaging (MRI) plays an important role in diagnosis by demonstrating neurovascular compression of the medulla and excluding other causes of neurological deficits such as infarction, demyelination, neoplasm, or inflammatory pathology [2,4]. However, vertebral artery indentation of the medulla may also be encountered incidentally in asymptomatic individuals, making clinical correlation essential for establishing the diagnosis [1,2,6].

We present two cases of vertebral artery compression of the medulla demonstrating different clinico-radiological scenarios: one patient with isolated symptomatic vascular compression and another with coexisting acute ischemic infarcts, highlighting the diagnostic challenge of symptom attribution in patients with concurrent cerebrovascular disease.

## Case presentation

Case 1

A 77-year-old woman presented with acute-onset left-sided hemiparesis associated with gait imbalance and dysarthria, with clinical suspicion of a cerebrovascular accident. Neurological examination revealed left-sided upper motor neuron weakness, and mild cerebellar ataxia and impaired coordination were also noted. MRI demonstrated multiple areas of T2/fluid-attenuated inversion recovery (FLAIR) hyperintensity showing diffusion restriction and corresponding low apparent diffusion coefficient values involving the right insular and occipital cortex and right corona radiata, consistent with acute non-hemorrhagic infarcts. Additional chronic ischemic changes and chronic lacunar infarcts were noted (Figure 1).

**Figure 1:**
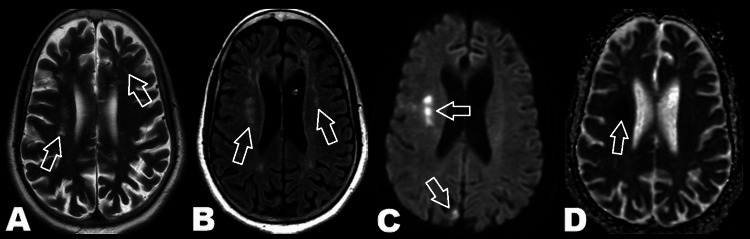
Neurovascular findings of acute stroke and chronic ischemic changes. Axial T2 (A) and T2 FLAIR (B) images show hyperintensities in bilateral periventricular regions - ischemic changes.  Axial DWI (C) and corresponding ADC map (D) show true diffusion restriction in the right periventricular region and occipital cortex - acute infarcts. FLAIR: Fluid-attenuated inversion recovery; DWI: diffusion weighted imaging; ADC: apparent diffusion coefficient

The posterior fossa on plain and MR angiography images revealed a tortuous and mildly ectatic left vertebral artery, causing focal compression of the left anterolateral aspect of the medulla. T2-weighted cisternography sequence (CUBE) also demonstrated the arterial course and medullary compression with multiplanar reformats (Figure 2). No hemodynamically significant stenosis, aneurysm, or arteriovenous malformation was identified.

**Figure 2:**
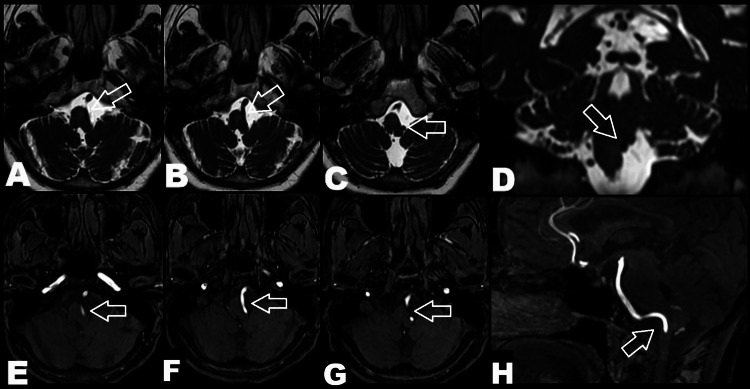
Compression of the ventrolateral medulla by the tortuous left vertebral artery. Axial T2 CUBE cisternography images (A to C) show a tortuous left vertebral artery compressing the ventrolateral aspect of medulla oblongata and displacing it right laterally. Coronal reformatted T2 CUBE cisternography image (D) better delineates the focal indentation of the medulla. Axial MR time-of-flight (TOF) angiography images (E to G) and Sagittal reformatted maximum intensity projection (MIP) image (H) demonstrate the tortuous and ectatic left vertebral artery. The right vertebral artery is relatively narrow in caliber but with maintained flow.

Imaging findings were consistent with cerebrovascular ischemic disease with multiple right-sided acute infarcts along with incidentally detected vertebral artery compression of the medulla.

Although the patient had imaging evidence of VAMCS, concurrent ischemic infarcts represented a significant confounding factor for clinical symptom attribution.

Case 2

A 46-year-old man presented with weakness involving the right upper and lower limbs, gait imbalance and ataxia for approximately seven years. Neurological examination revealed long tract signs of hyperreflexia and mildly increased tone on the right side. Magnetic resonance imaging of the brain revealed a prominent left vertebral artery and posterior inferior cerebellar artery (PICA) branch causing focal compression and distortion of the left anterolateral aspect of medulla oblongata without associated abnormal intramedullary signal intensity (Figure 3). No hemodynamically significant stenosis, aneurysm, or arteriovenous malformation was identified.

**Figure 3:**
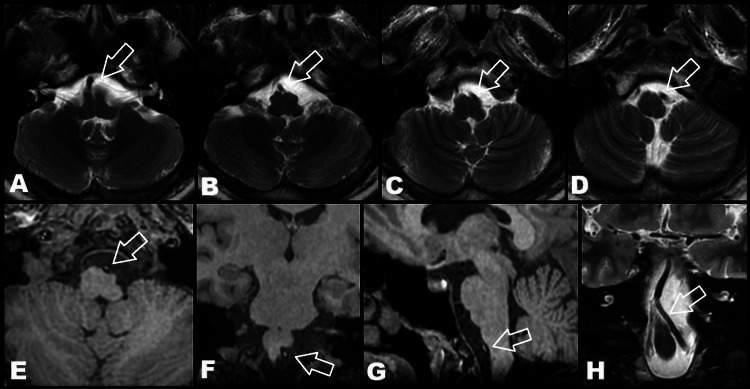
Compression of the ventrolateral medulla by the tortuous left vertebral artery. Axial T2 images (A to D) show a tortuous dominant left vertebral artery compressing the ventrolateral aspect of the medulla oblongata and mildly displacing it right laterally. The small vessel posterior to the left vertebral artery in (D) is the posterior inferior cerebellar artery branch, which also causes focal compression at that level along with the left vertebral artery. Axial (E), coronal reformatted (F), and sagittal reformatted (G) T1 gradient echo (FSPGR) images show flow-related enhancement of the left vertebral artery and focal indentation on the ventrolateral medulla. The coronal oblique T2 image along the plane of the vertebral artery (H) demonstrates well the tortuous course of the left vertebral artery.

Additional imaging findings included mild generalized cerebral and cerebellar atrophy and mild chronic small vessel ischemic changes. No acute infarct, intracranial hemorrhage, space-occupying lesion or other posterior fossa abnormality was identified.

In view of the focal vertebral artery-induced distortion of the medulla in the absence of other significant posterior fossa pathology, the imaging morphology suggests vertebral artery compression of the medulla syndrome. A definitive causal relationship would require post-surgical assessment; however, the patient was put on conservative management initially.

## Discussion

Vertebral artery compression of the medulla oblongata represents an uncommon but increasingly recognized neurovascular compression syndrome caused by tortuous, elongated, or ectatic vertebral arteries indenting the ventrolateral or anterolateral medulla. Although vertebrobasilar dolichoectasia is a known vascular abnormality, symptomatic medullary compression remains rare and likely underdiagnosed because the clinical manifestations are heterogeneous and frequently overlap with more common neurological disorders including posterior circulation ischemia, demyelinating disease, degenerative cervical myelopathy, and vestibulocerebellar disorders [1-3]. The present cases illustrate two important clinico-radiological scenarios encountered in VAMCS: isolated medullary neurovascular compression and medullary compression occurring in the setting of concurrent ischemic cerebrovascular disease.

The diagnosis of VAMCS is challenging because radiological vascular indentation of the medulla may also be encountered incidentally in asymptomatic individuals. Prior studies have emphasized that imaging evidence of neurovascular contact alone is insufficient for diagnosis and that careful clinical correlation is essential before attributing symptoms to vertebral artery compression [2,4]. In the first case, the presence of acute and chronic infarcts represented a major confounding factor, making it difficult to determine the exact contribution of medullary compression to the patient’s neurological manifestations. In contrast, the second case demonstrated focal distortion of the medulla by a prominent vertebral artery and PICA branch without significant pathology, representing a prototypical imaging appearance of isolated VAMCS.

Symptoms reported in the literature depend on the exact site of medullary compression and involved neural structures. Compression of the anterolateral medulla may affect corticospinal tracts, spinothalamic pathways, vestibular nuclei, nucleus ambiguus, and inferior cerebellar connections, resulting in combinations of vertigo, imbalance, gait ataxia, pyramidal tract signs, hemiparesis, hemisensory deficits, dysphagia, dysarthria, and lower cranial nerve dysfunction [2,5]. A review of surgically treated VAMCS cases has shown that vestibulocerebellar symptoms and pyramidal tract manifestations constitute the most frequent clinical presentations [3,5]. The literature further suggests that symptoms may occur either due to direct mechanical compression or secondary vascular insufficiency involving perforating vessels supplying the medulla [2].

MRI plays a central role in diagnosis by directly demonstrating the neurovascular relationship and excluding alternative pathologies. High-resolution T2-weighted sequences and heavily T2-weighted cisternography sequences such as CUBE, CISS, or FIESTA are useful for delineating focal medullary indentation and vascular loops. MR angiography and CT angiography further aid in demonstrating vertebral artery anatomy, dominance, tortuosity, dolichoectasia, or associated vascular anomalies [1-3]. In both cases, MRI clearly demonstrated focal distortion of the medulla by the dominant left vertebral artery. Notably, no intrinsic signal abnormality was identified within the medulla in both patients, a finding that has also been described in several prior reports [2,5].

Management of VAMCS remains individualized because of the rarity of the condition and absence of standardized treatment guidelines. Conservative management may be considered in mildly symptomatic patients or in cases where symptom attribution remains uncertain. Surgical treatment using microvascular decompression and vertebral artery repositioning techniques can have favorable outcomes in selected symptomatic patients with progressive neurological deficits [3,5]. Vertebral artery transposition or sling techniques may provide better long-term decompression compared with simple interposition procedures [5,6]. However, surgical decision-making remains technically challenging because of the position, large caliber, stiffness, and perforator-rich anatomy of the vertebral artery.

The present cases highlight the broad clinic-radiological spectrum of VAMCS and reinforce the importance of meticulous imaging review in patients with unexplained posterior fossa or long-tract neurological symptoms. They also emphasize the diagnostic difficulty of establishing symptom causality when vascular compression coexists with ischemic cerebrovascular disease.

## Conclusions

VAMCS is an uncommon neurovascular entity that may be considered in selected patients presenting with unexplained vestibulocerebellar symptoms, long tract signs, bulbar manifestations, or other posterior fossa neurological deficits. MRI plays an important role by demonstrating focal neurovascular compression of the medulla by a tortuous and ectatic vertebral artery while excluding alternative pathologies such as infarction, demyelination, or neoplasm.

The present cases illustrate the variable spectrum associated with vertebral artery-medullary contact, ranging from apparently isolated symptomatic compression to concurrent cerebrovascular disease, where direct symptom attribution remains difficult. Correlation with other neurological lesions and clinical findings is essential to differentiate incidental radiological indentation of the medulla versus clinically significant compression, thus guiding management.

## References

[1] Li Q, Xie P, Yang WS, Yan B, Davis S, Caplan LR (2019). Vertebral artery compression syndrome. Front Neurol.

[2] Savitz SI, Ronthal M, Caplan LR (2006). Vertebral artery compression of the medulla. Arch Neurol.

[3] Lombarski L, Kunert P, Skawiński M, Prokopienko M, Lewandowski Z, Marchel A (2018). Symptomatic medulla compression by vertebral artery. Neurol Neurochir Pol.

[4] Hongo K, Nakagawa H, Morota N, Isobe M (1999). Vascular compression of the medulla oblongata by the vertebral artery: report of two cases. Neurosurgery.

[5] Kim P, Ishijima B, Takahashi H, Shimizu H, Yokochi M (1985). Hemiparesis caused by vertebral artery compression of the medulla oblongata. Case report. J Neurosurg.

[6] Cierpiol S, Schäfer S, Gossner J (2015). Compression of the medulla oblongata due to an elongated vertebral artery is a common incidental finding on MRI of the brain. Acta Neurol Belg.

